# Hybrid Bayesian Network-Based Modeling: COVID-19-Pneumonia Case

**DOI:** 10.3390/jpm12081325

**Published:** 2022-08-17

**Authors:** Ilia Vladislavovich Derevitskii, Nikita Dmitrievich Mramorov, Simon Dmitrievich Usoltsev, Sergey V. Kovalchuk

**Affiliations:** National Center for Cognitive Research, ITMO University, 199034 Saint-Petersburg, Russia

**Keywords:** COVID-19, pneumonia, dynamical Bayesian networks, treatment trajectories, auto ML

## Abstract

The primary goal of this paper is to develop an approach for predicting important clinical indicators, which can be used to improve treatment. Using mathematical predictive modeling algorithms, we examined the course of COVID-19-based pneumonia (CP) with inpatient treatment. Algorithms used include dynamic and ordinary Bayesian networks (OBN and DBN), popular ML algorithms, the state-of-the-art auto ML approach and our new hybrid method based on DBN and auto ML approaches. Predictive targets include treatment outcomes, length of stay, dynamics of disease severity indicators, and facts of prescribed drugs for different time intervals of observation. Models are validated using expert knowledge, current clinical recommendations, preceding research and classic predictive metrics. The characteristics of the best models are as follows: MAE of 3.6 days of predicting LOS (DBN plus FEDOT auto ML framework), 0.87 accuracy of predicting treatment outcome (OBN); 0.98 F1 score for predicting facts of prescribed drug (DBN). Moreover, the advantage of the proposed approach is Bayesian network-based interpretability, which is very important in the medical field. After the validation of other CP datasets for other hospitals, the proposed models can be used as part of the decision support systems for improving COVID-19-based pneumonia treatment. Another important finding is the significant differences between COVID-19 and non-COVID-19 pneumonia.

## 1. Introduction

### 1.1. Background

The COVID-19 pandemic has affected the world for over two years. According to [1], over six million people have died and almost half a billion people have been infected during the pandemic. New mutated strains, such as the Omicron variant, spread around the globe and produce an immune escape, with a higher risk of reinfection than the Beta and Delta variants. It not only invades the respiratory system but also causes other organ injuries in severe cases, such as kidney injury, liver injury, myocardial injury, coagulation dysfunction, and gastrointestinal symptoms [2].

Symptoms of respiratory system failure are highly frequent in COVID-19 cases [3]. Over 15% of hospitalized COVID-19 patients develop acute respiratory distress syndrome (ARDS). The mortality of the critically ill group of COVID-19 patients is comparable with that of severe ARDS, reaching approximately 40% at day 30 after admission to the intensive care unit [4]. Most frequent reasons of ARDS are different types of pneumonia [5]. Therefore, the research of COVID-19 pneumonia is an essential and urgent task.

A disease trajectory modeling approach might be used for proper COVID-19 pneumonia diagnosis and treatment. Kim et al. claim that monitoring the early trajectory of pneumonia extent on chest radiographs could further stratify patients at risk for worse outcomes beyond the baseline tests [6]. Other studies provide new ideas to understand the heterogeneous etiopathology of COVID-19 patients and the associations of distinct trajectories with disease severity, which is essential to improve early risk assessment, patient monitoring, and follow-up schedules [7].

The outlined studies underscore the necessity of pneumonia disease trajectory modeling. In this article, we propose our own approach, using both ordinary and dynamic Bayesian Networks, but also modern auto ML approaches.

### 1.2. Related Works

There are many methods described in the literature that have been used for predictive modeling of disease trajectory. The most frequent approaches are hidden Markov models (HMM), deep learning, expert-based modeling, and Bayesian networks (BN). Hereinafter, we call these approaches as A, B, C, and D, respectively.

Approach A is a standard tool for disease modeling [8,9]. Yusuf A Amrulloh et al. presented a cough model based on the HMM. With data collected from a pediatric population diagnosed as pneumonia and asthma, the model achieved an accuracy of 82.7% for a pneumonia prediction task [8]. Ozonoff et al. proposed an approach based on HMMs, and demonstrated it on pneumonia and influenza (P&I) mortality data [9]. The paper shows that hidden Markov models are intuitively motivated and demonstrate improvements in goodness-of-fit, when applied to retrospective P&I mortality data. However, approach A does not consider “past” states of variables and relies on the current state of the presented variable. Furthermore, this approach cannot provide an approximation of difficult relationships between medical indicators.

Unlike approach A, deep learning approach B uses more sophisticated algorithms for constructing the model [10,11,12]. Artificial neural networks (ANN) have been used for pneumonia predictions [10]. Using a backpropagation algorithm, the feedforward ANN was trained on sociodemographic, symptom, sign, comorbidity, and radiographic outcome data. Among adults suffering from acute respiratory disease, the model accurately discovered patients with and without pneumonia [11] in a retrospective study for testing the ability of a deep learning algorithm at extracting features from chest x-rays (CXR) to track and predict radiological evolution. CXR deep learning features showed promise for classifying disease trajectory and may inform triage decisions after validation. CXR data have been used differently by Hoon Ko et al. [12], to develop an artificial intelligence technique to diagnose COVID-19 pneumonia in CT images and differentiate it from non-COVID-19 pneumonia and non-pneumonia diseases. Their fast-track COVID-19 classification network (FCONet), a simple 2D deep learning framework based on a single chest CT image, provided excellent diagnostic performance in detecting COVID-19 pneumonia. Despite the great performance of the deep learning approach in a large variety of tasks, it lacks interpretability, which is an essential part of disease modeling.

This disadvantage was eliminated by approach C, which nowadays is frequently used together with approach D [13,14]. For example, Shaochong Lin et al. aimed to develop a machine-learning model that identifies future high-cost patients with chronic obstructive pulmonary disease (COPD) and to ensure that such a model incorporates expert knowledge about causal relationships [13]. The learning BN structure was later used for model creation and showed considerable improvement compared with the baseline machine-learning methods. An application of expert knowledge towards learning BN structure is covered in [14]. Using multiple kinds of expert knowledge, it was shown that such an approach facilitates the knowledge engineering process and allows us to perform hybrid structure learning algorithms. Expert-based modeling could be used as a starting point of research or as a facilitation for some other methods, such as approach B. However, it has many limitations, such as the frequent lack of sufficiently evidence-based conclusions and statistically confirmed patterns of disease development [15].

Approach D is widely used in studies on COVID-19. Bayesian networks are known for underlying causal assumptions and interpretability and are widely used in disease modeling. For example, in our previous work, we compared BN and HMM built on COVID-19 pneumonia patients′ clinical data [16].

Nowadays, the continuous time Bayesian network (CTBN) and dynamic Bayesian network models have become an object of interest for many researchers in disease modeling. For example, Gatti et al. created a CTBN that was used to diagnose cardiogenic heart failure and to anticipate its likely evolution [17]. The proposed model managed to overcome the strong modeling and computational limitations of dynamic Bayesian networks (DBN) and allowed the direct representation of time, offering valid computational machinery for medical inference. For COVID-19 pneumonia, we propose a new approach, which facilitates the advantages of time period modeling and Bayesian networks and allows us to build complex and more realistic models for a COVID-19 pneumonia disease trajectory prediction task.

### 1.3. The Research Question

In the Introduction, we described reasons for researching COVID-19-pneumonia. In a short version of this paper, we investigated differences between CP and ordinary pneumonia [16]. Using these differences, we created models for automatically distinguishing different types of pneumonia. This model showed a prediction quality of more than 85 for the F1 score (best model: hidden Markov model, with 95% F1 score on the test samples). After validations using other COVID-19 datasets, it could be deployed in practice. However, using this model, we cannot improve the treatment process. These models do not support finding an optimal treatment strategy for new patients. Thus, we need to upgrade our approach.

To find an optimal treatment strategy for new CP cases, medical specialists need high-quality predictions about the future dynamics of important patients′ condition indicators. Here and below, we refer to predictions with a quality of more than 0.85 in terms of F1 score or accuracy on cross-validation for the test samples (that were not used for training models) as “high-quality predictions” and “high-quality models”.

These predictions should consider specifics of each treatment case and use all important history for the approximation of future indicators. Predictions should include treatment results, length of stay (for optimal planning of hospitals resources, which is especially important during a pandemic), and dynamics of all condition indicators as time-sequences for early prevention of critical conditions.

For solving the problem, we can use methods listed in the section Related Work. HMM models, according to approach A, are high-interpretations tools [18]. However, they cannot investigate the complex relationships between medical indicators. Neural networks, according to approach B, cannot be used, because predictions by this tool are hard to interpret. Moreover, validations using expert knowledge could be difficult. The literature overview on approaches B and C, as well as our experience with real COVID-19 data, show that Bayesian networks can make approximations with high enough quality (92% precision in a task of defining type of pneumonia [16]) of statistical influence from patients’ conditions to the targets.

Specifics of real data have many gaps for important indicators. For aggregated data, we divided the length of stay into several time intervals and aggregated values from this interval (the method is described in Section 2.1 and Section 2.2). We used a set of ordinary BN to find statistical relationships between the indicators from each treatment time interval. Then, we deployed DBN to find relationships between the indicators from different intervals and to predict all the values for the next interval using the last interval. Finally, we applied hybrid methods (auto ML and DBN together) for improving the prediction quality. Thus, we can predict full future sequences of values for each medical indicator using only information from the first interval.

## 2. Materials and Methods

**Figure 1:**
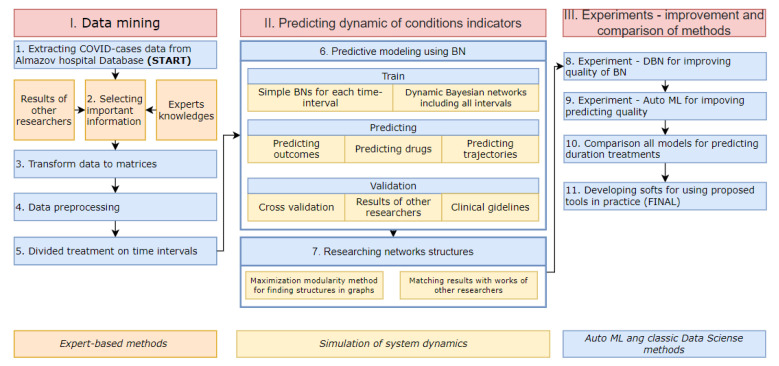
Procedure. Research includes 3 stages and 11 steps.

Figure 1 shows a chart of the procedure.

The first stage is data mining. In the first step, 6,302,049 electronic medical records from 2445 cases of COVID-19-based pneumonia were extracted from the Almazov Medical Research Database. The data are described in detail in Section 2.3. In the second step, we selected important features, using knowledge from the following three sources: clinical recommendations, experts′ knowledge, and works of other researchers. We selected 160 medicals indicators that are important in predicting treatment dynamics and treatment outcomes. Then, we performed data pre-processing. To overcome the problem of gaps, we used the MICE algorithm [19]. For coding categorical features, we applied the one-hot-encoding method. In the fifth step, we divided each treatment interval into several time intervals. Then, information for each interval of several days was aggregated and each interval was considered as a discrete time point. Patients′ conditions do not change every day, and for high-quality modeling of the trajectory of changing important indicators (in terms of metrics and experts′ opinion), we need aggregated time points.

In the second stage, patient condition indicators′ dynamics were predicted; this includes steps 6–7. In the 6th step, we trained OBN and DBN models using real data. These steps are described in detail in Section 2.1 and Section 2.2. Thereafter, we investigated statistical relationships between the indicators at each time interval using OBN. Furthermore, we tried using these networks to predict the treatment results. Then, we researched probabilistic relationships between the indicators at different time intervals using DBN. Based on trained DBN, we created tools for predicting sequences of all important indicators and treatment duration. Indicators included therapy. Therefore, we can predict future therapy effects in terms of changing important indicators for all time intervals. We validated models using classical metrics for predictive tasks and results of other researchers.

The third step included experiments for comparing and improving models from the second step. In step 8, we created DBN for improving prediction quality by ordinary BN for some time intervals. In step 9, we used the modern auto ML method for predicting length of stay (LOS) for CP-patients in hospitals and used a hybrid of the auto ML method and DBN approaches to achieve better predictive quality. Then, we compared methods (step 10) using cross-validation and classical metrics. Finally, we developed software for using the proposed models as part of medical decision support systems.

### 2.1. Simple Bayesian Networks

A BN is a directed acyclic graph, whose nodes represent random variables, and links express dependencies between the nodes. Each node is a random variable, i.e., $N_{i}\in N\left(1\leq i\leq n\right)$. Each edge is a link, E⊆N×N, and P is joint probability distribution, described as

(1) $$P\left(N\right)=\underset{N_{i}\in N}{\prod }P(N_{i}|\pi \left(N_{i}\right))$$

where $\pi (N_{i}$) is the set of parent nodes of $N_{i}$. In this article, we use hybrid Bayesian networks that contain both discrete variables and continuous variables [20].

Training Bayesian networks is a process of estimating parameters P that best represent the given data set D and creating a Bayesian directed acyclic graph (DAG). Several quality metrics of Bayesian networks exist, such as Bayesian information criterion (BIC) [21], maximum description length (MDL) [22], or Akaike information criterion (AIC) [23]. One of the most common metric of quality for created BN is log-likelihood (LL) [24], which is as follows:

(2) $$\mathrm{LL}(B|D)=\underset{N_{i}}{\sum }\log(P(N_{i}|P(N_{i}|\pi_{B}\left(N_{i}\right))))$$

where B is the Bayesian network over D, and $|\pi_{B}\left(N_{i}\right)\;$is the parent node of $N_{i}$ in B.

This metric is often used for creating predictive Bayesian networks in medical tasks [25,26] and results in high performance of the created models. For example, in the paper [25], BN showed 0.89 AUC-ROC quality in a task of medical classification. This result was better than the results of a logistic regression model and naive Bayesian classifier. In task [26], the Bayesian network and regression method were implicated in treatment cost prediction. This model showed 89.14 accuracy, which is better than the second result (by locally weighted LASSO regression model) by 4%.

Furthermore, the authors empirically evaluated the capability of various scoring functions of Bayesian networks for recovering true underlying structures [22]. Z. Liu and colleagues explained that MLD and BIC methods consistently outperform other scoring functions, such as Akaike’s information criterion (AIC), Bayesian Dirichlet equivalence score (BDeu), and factorized normalized maximum likelihood (fNML). In the current paper, we use BIC to find the optimal DAG structure for Bayesian dynamical networks.

We train Bayesian networks to carry out the following tasks:

- Find statistical patterns between patients′ condition indicators.
- Select dynamical predictors (indicators and time of its measurement) of treatment outcomes.
- Predict treatment outcomes.

The algorithm for training the network includes the following five steps:

1. The dataset is separated by 5 time periods—each period lasts 7 days. Patients′ conditions do not change every day. For researching statistically significant patterns, we, therefore, aggregate information for each interval.
2. Learning the DAG. There are two approaches for finding the structure of the BN graph, score-based structure learning algorithms and constraint-based algorithms. We try to use two methods from the first approach—hillclimb search and Chow-liu and one method from the second approach—constraint-based search [27]. Hillclimb search performs a greedy local search that begins with default DAG without edges and proceeds by iteratively performing single-edge manipulations that maximally increase the score (BIC). Chow-liu is based on the hypothesis that networks have a tree structure. The tree structure shows the best metric, given that each node has at most one parent. The constraint-based approach identifies probabilistic dependencies in the data set based on hypothesis tests, such as chi-square.
3. Learning the network parameters using the maximum likelihood estimation method [28].
4. Visualizing Bayesian networks as graphs. Nodes were clustered using the modularity maximization method. This method is based on the widely-used objective function to determine communities from a given network [29].
5. Estimating the predictive quality of each network in terms of predicting treatment outcomes by using the classical metric of predictive tasks, including F1 score for the classification problem and mean absolute error (MSE) for regression.

### 2.2. Dynamic Bayesian Networks

**Figure 2:**
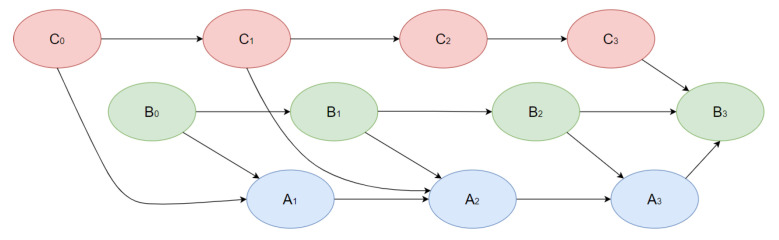
DAG-example of a dynamic Bayesian network.

In this research, we went beyond ordinary Bayesian networks by using dynamical Bayesian networks (DBN). A DBN is a Bayesian network that relates variables to one another over adjacent time steps. Figure 2 demonstrates the visualization of an example DBN, which includes three states A, B, C and four time periods (0–3). By states, we mean any components of an object (patient) condition in a dynamical discrete system, which may be related to one another within one-time intervals and between different time intervals, as shown in Figure 2. Processes of learning parameters and structures, as well as making inferences, are similar to ordinary BN. The structure of DBN is described in more detail in [30].

In this paper, we use DBN for the following tasks:

- Research of dynamical statistical patterns of treatment trajectories.
- Predicting the full future trajectory of important patients′ indicators
- Predicting the therapy effect in terms of treatment outcomes and length of stay (therapy included in factors of networks)

**Figure 3:**
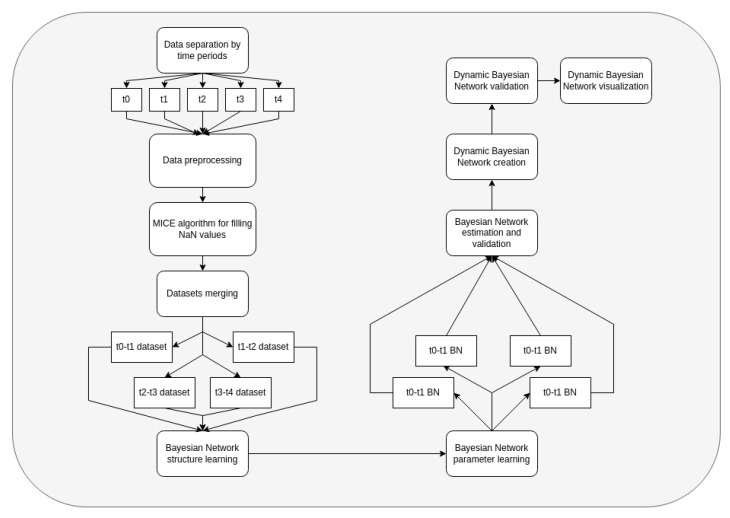
Dynamic Bayesian network modeling algorithm.

The algorithm for training DBN is described in the chart below (Figure 3).

The algorithm comprises the following steps:

1. The dataset is separated into 5 time periods, each of them with a 7-day length.
2. The MICE algorithm is applied to fill the missing data, since real clinical data might contain missing values. Features without data are dropped.
3. Information from day t to day t +1 is merged and used for the creation of Bayesian networks. Data are separated into train and test datasets for every time.
4. Learning DAG and parameters of joint probability distribution are similar to learning for ordinary BN (algorithm in the item 2.2). All networks learn using the BAMT python package [31,32].
5. We validate DBN using only test samples. Using models, we made predictions of the treatment outcomes and a series of important patient condition indicators using the following algorithm: using initial data from t-0 interval, we provide predictions of all the medical indicators for t-1 interval; using predicted indicators, we make predictions for the t-2-time interval, and so on. When we have predictions of the patients′ condition indicators, we predict the treatment outcomes.
6. For analyzing structures of trained DBN, we visualize it as a graph; we deploy the method of modularity maximization to divide networks into clusters and to analyze the model’s structure.

### 2.3. Data

The study is based on a dataset including 6,302,049 medical records for 2445 patients who were treated for COVID-19 pneumonia at the Almazov National Medical Research Centre, St. Petersburg, Russia, in 2020–2021. There are several entries and exclusion criteria for a patient to be included in dataset. The criteria are shown in Table 1.

For analysis, we used electronic medical records that fully describe each treatment cases. Information includes results of laboratory tests (blood, urine, cerebrospinal fluid, etc.); results of diagnostic procedures, such as CT scans of the lungs, x-rays, clinical parameters, such as heart rate, blood pressure, anthropometric parameters; symptoms; vaccination information; physical measurements; lifestyle; medication and many more. The time interval for each treatment case is described using a set of more than 160 indicators. Table 2 describes these data in more detail.

## 3. Results

### 3.1. Simple Bayesian Network-Based Analysis

**Figure 4:**
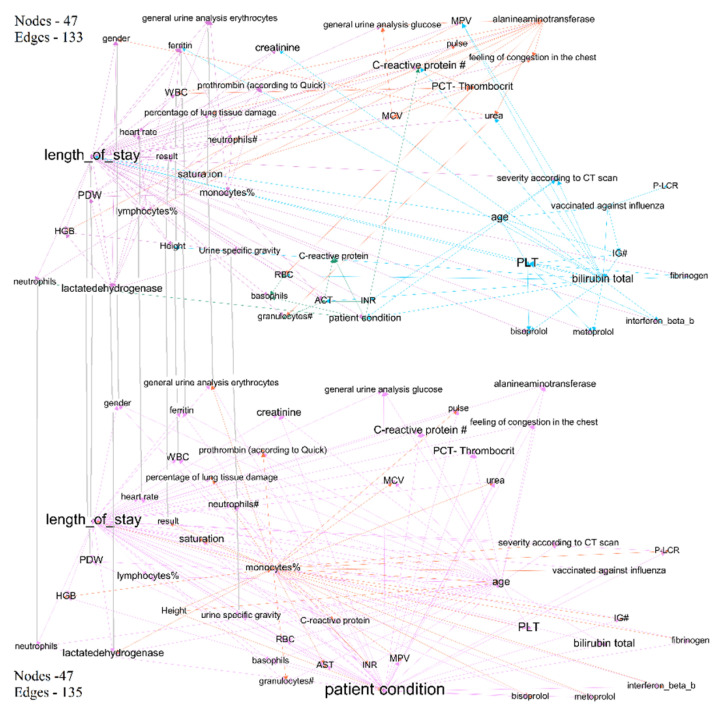
Graphs of trained BN for two-time intervals—first-time interval (top) and last-time interval (bottom). The colors represent nodes’ modularity maximization clustering. The size of each node is weighted degree.

We trained four BN (for each period) using the algorithm described in Section 2.1. One of the main goals of training BN is to research statistical relationships between medical indicators, and to analyze the dynamics of these relationships. For that purpose, we presented BN as graphs and applied the method of modularity maximization for analyzing the graph’s structure. We considered how the relationships and graph structure changed in the models for the first- and last-time interval (graphs in Figure 4).

We can observe that these two graphs have different structures. Table 3 describes those clusters and our conclusions in some detail.

**Figure 5:**
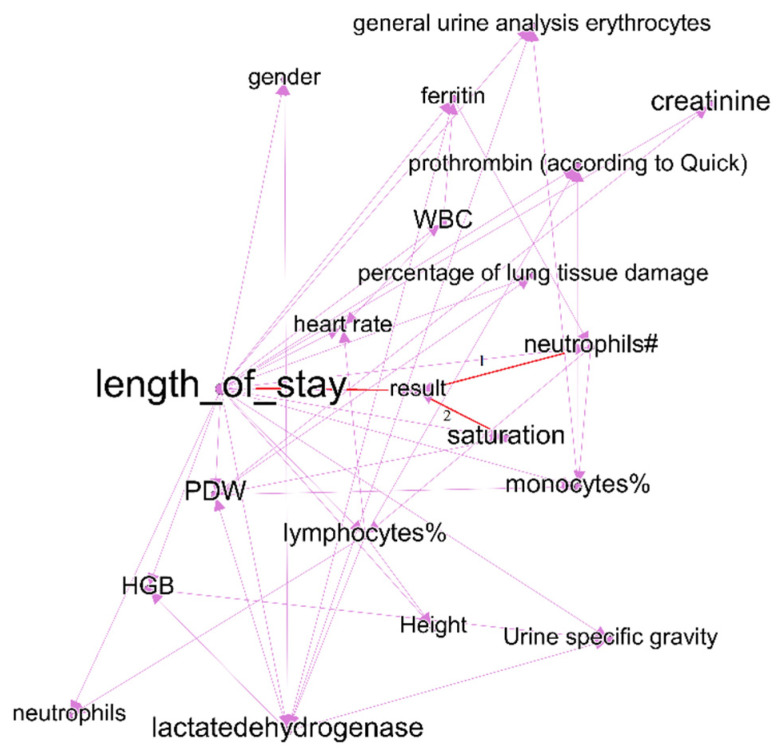
Cluster from DAG for the first-time interval, which includes the variable length of stay and treatment outcomes.

**Figure 6:**
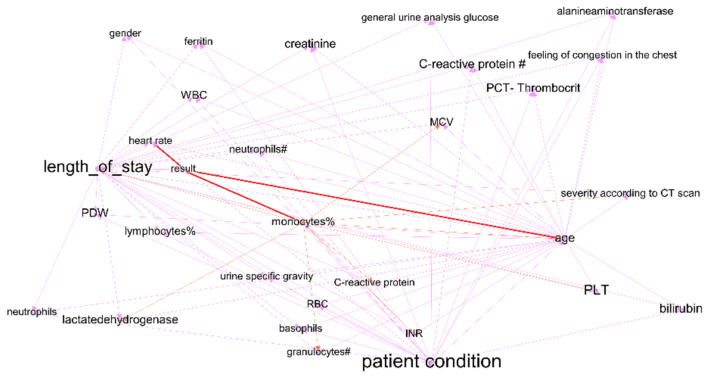
Cluster from DAG for fourth time interval, including length of stay and treatment results.

Bayesian network graphs show different relationships between indicators and different set of nodes with links to treatment outcomes for different time intervals. We also analyzed the graphs for other time intervals and made conclusions from the statistical relationships and their changes over time. The lists of dependencies and joint probability distributions are important findings for fundamental medicine. We analyzed clusters with treatment outcomes for first-time and last-time intervals in detail. Subgraphs are shown in Figure 5 and Figure 6.

The first target is the length of stay, which has links to all indicators of the purple clusters. These indicators include blood and urine laboratory results, level of saturation, percentage of lung tissue damage, heart rate, and gender. Other researchers confirmed parts of these relationships, e.g., the influence of lymphocytes [33] on treatment outcomes. Node results (the binary indicator of treatment outcome—fatal or recovered) have three links (marked red) to saturation (matched to the results of other researchers [34]), neutrophils (matched to results in [35]) and length of stay (the dependency is evident).

The purple cluster of the graph was based on medical indicators from the fourth time interval (from day 21 to day 29 of the patient’s stay in the hospital) and has many similarities with the cluster for the first interval. The length of stay was connected to all indicators of the clusters. Most indicators match for these two graphs, which demonstrates the stability and significance of the discovered relationships in the two graphs. The graph in Figure 6 shows a link between the length of stay and C-reactive protein (its link with COVID-19 outcome has been proven in many studies [36]) on the one hand and PLT (which influences COVID-19-related length of stay as described in [37]) on the other hand.

Similarly, predictors of treatment outcomes were identified for the graph of each interval. The statistical connections found, as well as some references to the work of other researchers who obtained similar results, are shown in Table 4.

Upon training, four BN were validated. For the test samples, we predicted treatment outcomes and estimated the predictive metrics. For the variable of treatment result, the metrics are accuracy and F1 score (classification problem). For the variable of length of stay, the metric is the mean absolute error (MAE). We state that the proposed hybrid Bayesian network can predict categorical and continuous variable simultaneously. In addition, its advantage in comparison to some ML models is that they solve only one type of task (classification or regression). To solve two tasks, we need to train two ML models. Table 5 shows the metrics for each interval and variables. All metrics have been calculated using cross validation with 5 folds.

The metrics show that the predictive quality of the treatment results increases from the t-0 to the t-2 interval. As for the t-3 interval, it decreases significantly. Many patients leave the hospital before the beginning of this interval (recovery or fatal outcome). Therefore, there were fewer precedents, which explains the deterioration in the quality of the prognosis.

**Figure 7:**
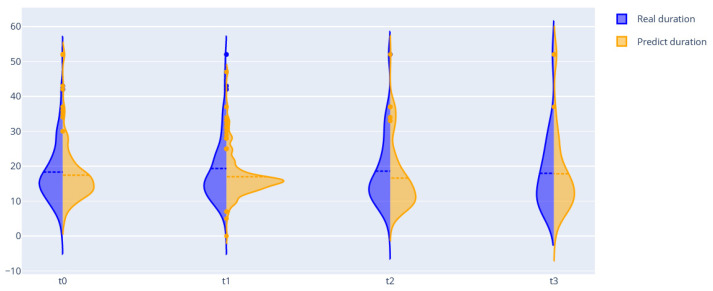
Real and predicted probability distributions of length of stay for predictions using information from different time intervals.

The quality of prediction of treatment outcomes is more than 0.8 (quality improves as new information becomes available, as observed in Table 5), for a length of stay MAE near one week. Figure 7 presents violin plots with real and predictive distributions of length of stay. We can observe that, for all intervals, the probability distribution is similar. Therefore, we can use this model for predicting the length of stay for a large patient population. However, the MAE metric is more than 10 for the t-3 intervals. For one patient, the error could be significant. We improve the quality for this interval using dynamical Bayesian networks further.

Using ordinary BN, we found statistical patterns and selected predictors for each treatment outcome for each time interval. Furthermore, we used information for creating more complex predictive models.

### 3.2. Dynamical Bayesian Network-Based Analysis

**Figure 8:**
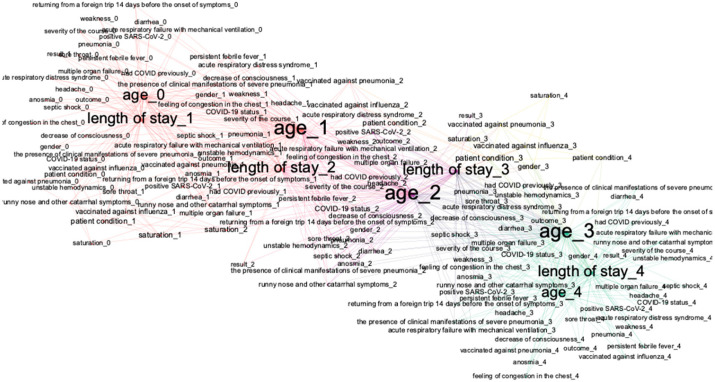
DAG for DBN for predicting treatment outcomes and length of stay.

For improving the prediction quality of length of stay, we developed dynamical Bayesian networks (DBN) that consider dynamical relationships between indicators and make better approximations than ordinary BN. Algorithms used for creating DBN are described in Section 2.2. We use the BIC metric for finding the DAG structure, and the log-likelihood metric for parameters learning. Furthermore, we use modern soft BAMT for working with DBN [32]. We selected features for DBN to be considered for detecting dependencies, as described in Section 3.1. The DAG of the created model is presented in Figure 8.

The results of modularity maximization clustering are four clusters within the graph. The investigation of the graph structure reveals that the model considers the age variable to be the most essential one for all patients and allows the consideration of a large variety of symptoms (including severe ones) every time. Using this network, we can predict length of stay. The quality of prediction is estimated using the MAE score. DBN improved the results of predicting the length of stay using information from the t-3 interval. DBN shows 8.12 MAE against 10.22 by using the simple Bayesian network from the 3.1 item. The major advantage of DBN is its good interpretability, as well as the increased confidence of doctors.

**Figure 9:**
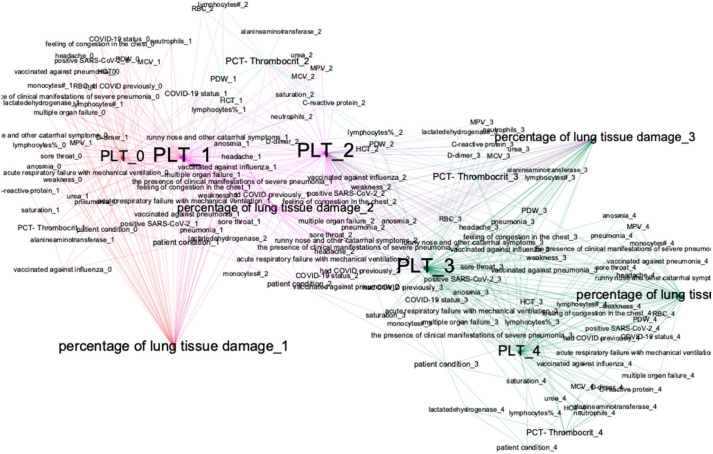
DBN for predicting time series of patient condition indicators.

Next, we created a separate DBN for predicting time series of variables that are important for treatment outcomes. We selected a set of variables based on the conclusions from Section 3.1. The created model has been visualized in a graph form, applying the modularity analysis to locate clusters and to obtain insights about the model’s inner structure (see Figure 9). This model uses information from the patients′ blood tests (such as PLT) and lung tissue damage data to predict a set of features, which would directly affect the future condition of the patient. Using initial data from blood tests at the t0 period and data about vaccination or general health state, it evaluates the severity of lung tissue damage for every period, mostly relying on blood test data.

**Figure 10:**
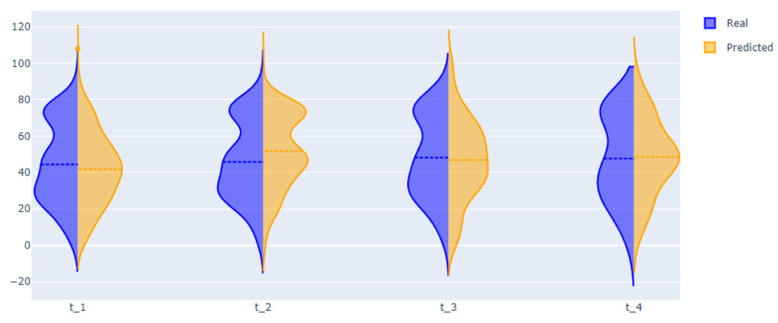
Real and predicted distribution of percentage of lung tissue damage.

**Figure 11:**
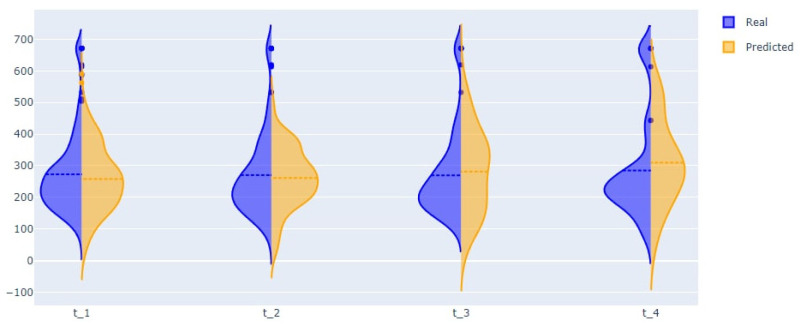
Real and predicted distribution of PCT.

The modularity analysis divided nodes into four clusters with different structures than those in the previous DAG. We trained networks and predicted all variables (from the graph) using only real values from the first time. The violin plots showed similarities in the probability distributions of the real and predicted variables (Figure 10 and Figure 11).

After validation using classic predictive metrics and comparisons of distributions, we concluded that the quality of the model is sufficient for deploying DBN in combination with auto ML methods to predict the length of stay (Section 3.3).

One of the main goals of this research is the prediction of future therapy trajectories for CP patients. Using the created DBN, we provided predictions of therapy trajectories in terms of sequences of prescribed CP drugs. The learning process is similar to the models described above. Nodes include a list of anti-CP-drugs that are most frequently applied in Russian hospitals at different time periods, including the following: ambroxol, dexamethasone, bisoprolol, azithromycin, etc. We assessed the therapy frequency in different intervals. Thereby, patients with and without applied therapy were in our training data for further estimation. Table 6 shows the quality metrics of proposed DBN.

**Figure 12:**
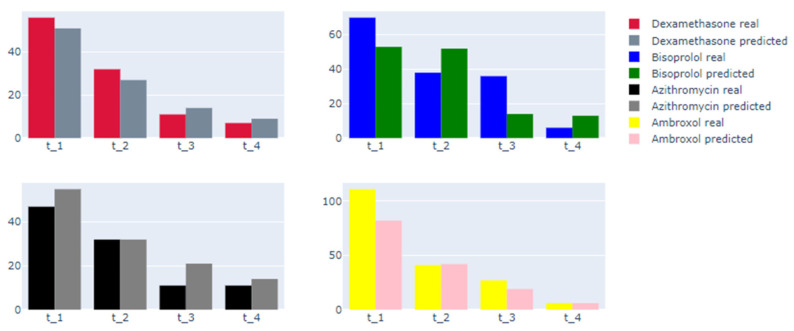
Comparison of real and predictive counts of drug prescribing.

The created DBN is capable of predicting therapy trajectories and combines both therapy and patient clinical data. The model enables the prediction of therapy trajectories every time (see Figure 12). We achieved the best overall f1-score (higher than 0.8) with the drug dexamethasone. For ambroxol, we observed the best score of 0.998 for the t4 period; however, metrics for the t1–t3 periods state that the model needs more fine-tuning to predict such therapy successfully and thoroughly. The model performed well with the azithromycin prediction. However, the prediction quality was significantly decreased for the last period. The root cause of this problem lies in the lack of training data for the later periods, which affects both the t3 and the t4 intervals.

From Table 6, with enough training data available, the model could provide decent scores, but therapy predictions for later periods were not as precise as for the t1 and t2 periods. However, with enough training data provided, the model increased its performance for the later time periods. The metrics for t1–t2 demonstrated good quality for approximations of relationships between patients′ condition indicators and prescribing therapy. After validation by medical specialists, this model could, therefore, be used for simulating the process of therapy prescribing, and for supporting decision-making in the COVID-19-pneumonia treatment process.

### 3.3. Hybrid Approach

In modern bioinformatics literature, authors often use various machine learning methods for creating predictive models. In specific practical settings, results of BN can upgrade the quality of ML models, and CP outcome prediction is one of these tasks. We provided such an outcome by using FEDOT [45] for predicting the length of stay and the output of DBN for improving the quality of FEDOT’s results. FEDOT is an open-source framework for automated modeling and machine learning (auto ML). It can build custom modeling pipelines for different real-world processes in an automated way using an evolutionary approach. FEDOT supports binary and multiclass classification, regression, clustering, and time series prediction tasks [46]. We deployed it for predicting the length of stay and compared results of the FEDOT method with the hybrid method, combining FEDOT and DBN to predict future series of medical indicators. The FEDOT algorithm includes the following steps: feature selection using the tree-based features importance method; developing the structure of FEDOT model, including modeling the process pipeline as sequences of the methods for preprocessing and prediction; cross-validations with the MAE metric. The hybrid method included just one extra step. Before we selected the features for each sample, we trained DBN, and we predicted the future time-series of indicators that were important in terms of treatment outcome predictions. For feature selection, we chose features from two sets, real indicators and predictions of future dynamics of treatment outcomes predictors (the feature list is provided in Section 3.1). That way, we expanded the dataset with new variables. Table 7 compares the quality of the following three methods: results of ordinary BNs (Section 3.1), FEDOT, and the hybrid method combining FEDOT and DBN.

**Figure 13:**
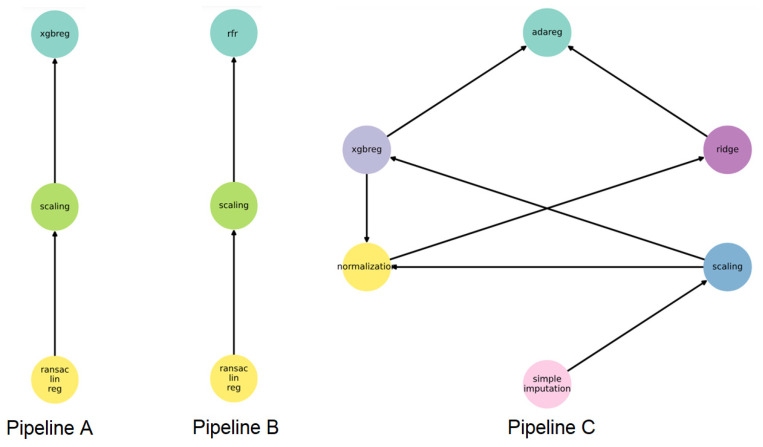
Pipelines of created predictive models for three-time intervals.

In three of the four intervals, the hybrid method combining FEDOT and DBN presents the best quality. Pipelines of the three best models are shown in Figure 13.

Pipeline A is used for the (t-0) and (t-1) intervals, pipeline B is used for the (t-2) intervals, and the last interval (t-3) is modeled by the more complex pipeline C. Each pipeline includes a data-scaling step. Pipelines for the three best models include a XGB regressor. The pipeline for the t-2 interval includes a random forest regressor instead of XGB. It could explain the deterioration in the quality of the hybrid method compared with conventional FEDOT. Metrics from Table 7 show that the approach provides a small error (MAE near 3 for all time intervals). The model could be useful for predicting the length of stay for patient flow in a hospital. It can support decision-making in optimization management of hospital resources, which is especially important in a pandemic.

## 4. Discussion

The outcomes of this paper are twofold. First, the research contributes to the evidence base of medicine with information about the course of COVID-19 pneumonia. Secondly, this paper proposes practical tools for predicting important indicators of future conditions of patients with COVID-19-based pneumonia and new algorithms (or new pipelines of using existed algorithms) for modelling the course of the disease in the hospital. The first outcome is presented in items 3.1–3.2. We suggested a Bayesian network-based method for identifying the following three types of probabilistic relationships: factor-to-factor within time interval, factor-to-factor between time interval, and factor-to-target. The extracted probabilistic relationships inform evidence-based knowledge on the course of COVID-19 pneumonia with inpatient treatment. Some of these relationships matched with the results of other researchers [38,39,40,41,42,43,44]. However, other extracted results are new, such as the influence of monocytes from the third time interval (10–17 days) to lethality. Our models show that this is one of the main predictors of treatment outcomes from this interval.

Moreover, the novelty of this research is the study of the course of COVID-19 pneumonia as a dynamic process using graph-probabilistic models. Probabilistic discrete-time models are often used for modelling dynamics of the COVID-19 epidemic [47,48], but almost never used to model the course of the disease in the hospital on the macrolevels. Abhinav Vepa and colleagues used Bayesian networks to extract probabilistic relationships and predict treatment outcomes [49]; however, their work does not research the course of the disease as a dynamic process with the analysis of all types of relationships.

The second outcome of the paper includes four approaches for predicting important disease indicators. Three approaches are based on sets of Bayesian networks, dynamic Bayesian networks, and the state-of-the-art auto ML framework FEDOT. The fourth approach is based on a new algorithm that uses the DBN and framework together. The quality of the developed tools for predicting CP length of stay is shown in Table 5 and Table 7. The best quality of cross-validation (mean for all test samples) was shown by the hybrid algorithm, which we associate with using temporal information extracted from data using the DBN, together with linear and nonlinear patterns extracted from data using auto ML. The results also include a tool to predict facts of different prescribed CP therapy drugs, which is a high-interpretability tool that supports the selection of therapy. Table 6 shows that for some drug models, the quality is more than 0.8 in terms of F1 score (e.g., dexamethasone), while for other drugs, the mean quality is less than 0.7 (e.g., azithromycin). This variability can be attributed to the different rigidity of rules for including drugs in the treatment strategy, and the consequently different difficulties of extracting this rule from data. In addition, we propose tools for predicting future multidimensional time series of patient condition indicators based on DBN. The violin plots in Figure 10 and Figure 11 show that the distributions of the predictive indicators are similar to the real distributions.

One of the main advantages of BN-based approaches is high interpretability. By “high-interpretability”, we mean that the predictions of the DBN algorithm are more understandable in comparison with other popular machine learning algorithms, such as neural networks, random forests and other. Each prediction of DBN can be interpreted using an understandable graph representation of causal probabilistic relationships between the indicators. For each case, we can extract an algorithm reasoning sequence using Bayesian probabilistic inference [50]. This increases the confidence of doctors and contributes to the implementation of the solution in practice.

## 5. Conclusions

In this paper, we proposed Bayesian network-based approaches for solving the following four problems: prediction of treatment outcomes for inpatient cases; prediction of the dynamics of patient conditions indicators; simulation of doctor’s therapy choice; upgrading the quality of the ML method for treatment outcome prediction.

All methods were applied on real practice tasks to optimize the COVID-19-based pneumonia treatment process. The developed practice tools have been validated using real cases that are different from those deployed in the model learning process, and they demonstrated high metrics of predictive quality (Table 5, Table 6 and Table 7). Furthermore, all the results have been matched with results of other researchers (Table 4) and modern clinical guidelines. In addition, dynamical Bayesian networks are a high-interpretability tool (“high interpretability” is explained in detail in the previous section), and this will increase the confidence of doctors and contribute to the implementation of the tool in practice.

Finally, we found statistical relationships in the dynamics of the COVID-19 pneumonia treatment process. High metrics of approximation (Table 5 for treatment outcomes and Table 7 for length of stay) demonstrates the reliability of these relationships. Thus, this paper contributes to evidence-based medicine and could be the basis for developing other models for CP patients.

The software developed for predictive modeling could be used as part of decision support systems in caring for COVID-19-based pneumonia patients.

Future work will include research on creating modules that include knowledge from fundamental medicine and developing a new hybrid approach, combining data-driven models and expert-based models for improving the quality of predictive modeling of treatment dynamics.

## Figures and Tables

**Table 1:** Entry and exclusion criteria for including a patient in the dataset.

Entry Criteria	Exclusion Criteria
1. COVID-19-based pneumonia 2. Minimum length of stay is three days 3. Treatment outcomes include lethal outcome and hospital discharge 4. Treatment process was inpatient	1. Observation period less than three days

**Table 2:** Medical indicators for treatment cases.

Feature’s Group	Features
Anthropometrics parameters	Height, weight, gender, body mass index, body surface area.
Simple measurements	Systolic blood pressures (SBP), diastolic blood pressure; (DBP), heart rate, temperature, saturation (SPO2), respiratory rate.
Laboratory results	101 indicators: different type of laboratory tests: venous and arterial blood tests, urine tests, cerebrospinal fluid tests, etc.
COVID-19 symptoms	Headache, unconsciousness, cough, sore throat, pus in the throat, feeling of congestion in the chest, type of breathing, weakness, decreased consciousness.
Results of diagnostic procedures	The presence of clinical manifestations of severe pneumonia, the percentage of lung tissue damage, the severity of the course, the patient’s condition, NEWS score, etc.
Vaccination	Flu, pneumonia, COVID-19 vaccination.
Complications	Multiple organ failure, septic shock, febrile fever, unstable hemodynamics.
COVID-19 therapy	Information of prescribed therapy for COVID-19 treatment drugs from clinical recommendations, including glucocorticosteroids, monoclonal antibodies, anticoagulants, antivirals, non-steroidal anti-inflammatory drugs and other drugs from current clinical recommendations.

**Table 3:** Description of the resulting network for the first-time interval.

Time Interval	Cluster	Description	Count of Nodes	Nodes with the Highest Power	Weighted Average Degree
First week of treatment	Purple	Includes predictors of treatment outcomes, and two outcome indicators—treatment outcomes and length of stay.	21	Duration of hospital stay (treatment outcome)	19
Fourth week of treatment	Purple	Includes predictors of treatment outcomes, and two outcome indicators—treatment results and length of stay.	28	Length of stay (treatment outcome)	10
First week of treatment	Orange	Some laboratory test results. Cluster does not include nodes with link to treatment outcomes.	4	PCT-plateletcrit	8
Fourth week of treatment	Orange	Some laboratory test results. Cluster includes nodes with link to treatment outcomes—monocyte% and saturation.	18	Saturation (link to treatment results)	8
First week of treatment	Green	C-reactive protein, RBC, basophils—indicators that have links to treatment outcomes (length of stay); however, they are not included in purple clusters. In the graph of the last period, this cluster joins with purple, part of indicators lose the link with treatment outcome.	12	C-reactive protein	10
First week of treatment	Blue	Patient condition and features that influence it—age, severity according to CT scan, bilirubin total, information of vaccination. In the fourth interval cluster, nodes transfer to purple cluster.	7	PLT	15

**Table 4:** Predictors of treatment outcome from four-time intervals.

Outcome Feature	t0	t1	t2	t3
Treatment outcomes	Saturation	Lymphocytes [38]	Monocytes	Age
Treatment outcomes	Neutrophils [39]	Red blood cells [40]	Age	Patient condition
Length of stay	Feeling of congestion in the chest	Urea	MCV mean corpuscular volume [41]	C-reactive protein
Length of stay	PCT-plateletcrit	MCV mean corpuscular volume [41]	Alanineaminotransferase [39]	Lactatedehydrogenase [42]
Length of stay	Neutrophils	C-reactive protein [43]	C-reactive protein	PDW—platelet distribution width [44]

**Table 5:** Metrics of BN models.

	t0	t1	t2	t3
Treatment outcomes—accuracy	0.87	0.94	0.95	0.84
Treatment outcomes—F1-score	0.84	0.93	0.95	0.82
Length of stay	8.61	7.3	9.18	10.22 (needs improvement)

**Table 6:** Metrics of DBN predictions facts of prescribing drugs in different time intervals.

	t-1	t-2	t-3	t-4
F1-score—dexamethasone	0.8421	0.8666	0.9	0.8333
F1-score—ambroxol	0.6516	0.6538	0.6136	0.998
F1-score—azithromycin	0.7481	0.8461	0.8421	0.4166
F1-score—bisoprolol	0.743	0.74	0.5133	0.909

**Table 7:** Metrics of three methods in the task of predicting length of stay.

Methods	t-0	t-1	t-2	t-3
Set of ordinary BNs	8.61	7.3	9.18	10.22 (8.12)
FEDOT framework	4.12	**2.81**	2.65	3.95
FEDOT + DBN	**3.6**	2.45	**2.88**	**3.39**

## Data Availability

Not applicable.

## References

[1] COVID Live—Coronavirus Statistics—Worldometer. https://www.worldometers.info/coronavirus/.

[2] Cummings M.J., Baldwin M.R., Abrams D., Jacobson S.D., Meyer B.J., Balough E.M., Aaron J.G., Claassen J., Rabbani L.R.E., Hastie J. (2020). Epidemiology, clinical course, and outcomes of critically ill adults with COVID-19 in New York City: A prospective cohort study. Lancet.

[3] Shi N., Huang C., Zhang Q., Shi C., Liu F., Song F., Hou Q., Shen J., Shan F., Su X. (2021). Longitudinal trajectories of pneumonia lesions and lymphocyte counts associated with disease severity among convalescent COVID-19 patients: A group-based multi-trajectory analysis. BMC Pulm. Med..

[4] Symptoms of Coronavirus: Early Signs, Serious Symptoms and More. https://www.webmd.com/lung/covid-19-symptoms#1.

[5] Bauer T.T., Ewig S., Rodloff A.C., Müller E.E. (2006). Acute respiratory distress syndrome and pneumonia: A comprehensive review of clinical data. Clin. Infect. Dis..

[6] Kim J.Y., Ji Jung K., Yoo S.J., Yoon S.H. (2021). Stratifying the early radiologic trajectory in dyspneic patients with COVID-19 pneumonia. PLoS ONE.

[7] Tian D., Sun Y., Xu H., Ye Q. (2022). The emergence and epidemic characteristics of the highly mutated SARS-CoV-2 Omicron variant. J. Med. Virol..

[8] Amrulloh Y.A., Triasih R., Setyati A. Hidden markov model of cough from pediatric patients with respiratory infections. Proceedings of the 2016 International Seminar on Application for Technology of Information and Communication (ISemantic).

[9] Ozonoff A., Sukpraprut S., Sebastiani P. (2006). Modeling seasonality of influenza with Hidden Markov Models. Proc. Am. Stat. Assoc..

[10] Heckerling P.S., Gerber B.S., Tape T.G., Wigton R.S. (2003). Prediction of community-acquired pneumonia using artificial neural networks. Med. Decis. Mak..

[11] Duchesne S., Gourdeau D., Archambault P., Chartrand-Lefebvre C., Dieumegarde L., Forghani R., Gagné C., Hains A., Hornstein D., Le H. (2020). Tracking and predicting COVID-19 radiological trajectory using deep learning on chest X-rays: Initial accuracy testing. medRxiv.

[12] Ko H., Chung H., Kang W.S., Kim K.W., Shin Y., Kang S.J., Lee J.H., Kim Y.J., Kim N.Y., Jung H. (2020). COVID-19 pneumonia diagnosis using a simple 2d deep learning framework with a single chest CT image: Model development and validation. J. Med. Internet Res..

[13] Lin S., Zhang Q., Chen F., Luo L., Chen L., Zhang W. (2019). Smooth Bayesian network model for the prediction of future high-cost patients with COPD. Int. J. Med. Inform..

[14] Julia Flores M., Nicholson A.E., Brunskill A., Korb K.B., Mascaro S. (2011). Incorporating expert knowledge when learning Bayesian network structure: A medical case study. Artif. Intell. Med..

[15] Derevitskii I.V., Savitskaya D.A., Babenko A.Y., Kovalchuk S.V. (2021). Hybrid predictive modelling: Thyrotoxic atrial fibrillation case. J. Comput. Sci..

[16] Mramorov N., Derevitskii I., Kovalchuk S. (2021). Predictive Modeling of COVID and non-COVID Pneumonia Trajectories. Stud. Health Technol. Inform..

[17] Gatti E., Luciani D., Stella F. (2012). A continuous time Bayesian network model for cardiogenic heart failure. Flex. Serv. Manuf. J..

[18] Awad M., Khanna R. (2015). Hidden Markov Model. Effic. Learn. Mach..

[19] van Buuren S., Groothuis-Oudshoorn K. (2011). Mice: Multivariate imputation by chained equations in R. J. Stat. Softw..

[20] Puga J.L., Krzywinski M., Altman N. (2015). Points of Significance: Bayesian networks. Nat. Methods.

[21] Schwarz G. (1978). Estimating the Dimension of a Model. Ann. Stat..

[22] Liu Z., Malone B., Yuan C. (2012). Empirical evaluation of scoring functions for Bayesian network model selection. BMC Bioinform..

[23] De Campos C.P., Ji Q. (2011). Efficient structure learning of Bayesian networks using constraints. J. Mach. Learn. Res..

[24] Park E., Chang H.J., Nam H.S. (2018). A Bayesian network model for predicting post-stroke outcomes with available risk factors. Front. Neurol..

[25] Van Der Gaag L.C., Renooij S., Feelders A., De Groote A., Eijkemans M.J.C., Broekmans F.J., Fauser B.C.J.M. (2009). Aligning bayesian network classifiers with medical contexts. Lecture Notes in Computer Science (Including Subseries Lecture Notes in Artificial Intelligence and Lecture Notes in Bioinformatics).

[26] Tong L.L., Gu J.B., Li J.J., Liu G.X., Jin S.W., Yan A.Y. (2021). Application of Bayesian network and regression method in treatment cost prediction. BMC Med. Inform. Decis. Mak..

[27] (2022). Package ‘bnlearn’ Type Package Title Bayesian Network Structure Learning, Parameter Learning and Inference. https://www.bnlearn.com/.

[28] Ji Z., Xia Q., Meng G. (2015). A Review of Parameter Learning Methods in Bayesian Network. Lecture Notes in Computer Science (Including Subseries Lecture Notes in Artificial Intelligence and Lecture Notes in Bioinformatics).

[29] Tsung C.K., Lee S.L., Ho H.J., Chou S.K. (2021). A modularity-maximization-based approach for detecting multi-communities in social networks. Ann. Oper. Res..

[30] Łupińska-Dubicka A. (2012). Modeling dynamical systems by means of dynamic Bayesian networks. Sci. Bull. Bialystok Univ. Technol. Inform..

[31] Bubnova A.V., Deeva I., Kalyuzhnaya A.V. (2021). MIxBN: Library for learning Bayesian networks from mixed data. Procedia Comput. Sci..

[32] ITMO-NSS-Team/BAMT: Repository of a Data Modeling and Analysis Tool Based on Bayesian Networks. https://github.com/ITMO-NSS-team/BAMT.

[33] Tan L., Kang X., Ji X., Li G., Wang Q., Li Y., Wang Q., Miao H. (2020). Validation of Predictors of Disease Severity and Outcomes in COVID-19 Patients: A Descriptive and Retrospective Study. Med.

[34] Mukhtar A., Rady A., Hasanin A., Lotfy A., El Adawy A., Hussein A., El-Hefnawy I., Hassan M., Mostafa H. (2021). Admission SpO2 and ROX index predict outcome in patients with COVID-19. Am. J. Emerg. Med..

[35] Zeng Z.Y., Feng S.D., Chen G.P., Wu J.N. (2021). Predictive value of the neutrophil to lymphocyte ratio for disease deterioration and serious adverse outcomes in patients with COVID-19: A prospective cohort study. BMC Infect. Dis..

[36] Lentner J., Adams T., Knutson V., Zeien S., Abbas H., Moosavi R., Manuel C., Wallace T., Harmon A., Waters R. (2021). C-reactive protein levels associated with COVID-19 outcomes in the United States. J. Osteopath. Med..

[37] Mahboub B., Bataineh M.T.A., Alshraideh H., Hamoudi R., Salameh L., Shamayleh A. (2021). Prediction of COVID-19 Hospital Length of Stay and Risk of Death Using Artificial Intelligence-Based Modeling. Front. Med..

[38] Lai K.-L., Hu F.-C., Wen F.-Y., Chen J.-J. (2021). Lymphocyte count is a universal predictor to the health status and outcomes of patients with coronavirus disease 2019 (COVID-19): A systematic review and meta-regression analysis. medRxiv.

[39] Zhao Y., Chen Q., Liu T., Luo P., Zhou Y., Liu M., Xiong B., Zhou F. (2021). Development and Validation of Predictors for the Survival of Patients With COVID-19 Based on Machine Learning. Front. Med..

[40] Ramachandran P., Gajendran M., Perisetti A., Elkholy K.O., Chakraborti A., Lippi G., Goyal H. (2022). Red Blood Cell Distribution Width in Hospitalized COVID-19 Patients. Front. Med..

[41] Kilercik M., Demirelce Ö., Serdar M.A., Mikailova P., Serteser M. (2021). A new haematocytometric index: Predicting severity and mortality risk value in COVID-19 patients. PLoS ONE.

[42] Henry B.M., Aggarwal G., Wong J., Benoit S., Vikse J., Plebani M., Lippi G. (2020). Lactate dehydrogenase levels predict coronavirus disease 2019 (COVID-19) severity and mortality: A pooled analysis. Am. J. Emerg. Med..

[43] Tomasiuk R., Dabrowski J., Smykiewicz J., Wiacek M. (2021). Predictors of COVID-19 Hospital Treatment Outcome. Int. J. Gen. Med..

[44] Bommenahalli Gowda S., Gosavi S., Ananda Rao A., Shastry S., Raj S.C., Menon S., Suresh A., Sharma A. (2021). Prognosis of COVID-19: Red Cell Distribution Width, Platelet Distribution Width, and C-Reactive Protein. Cureus.

[45] Nikitin N.O., Vychuzhanin P., Sarafanov M., Polonskaia I.S., Revin I., Barabanova I.V., Maximov G., Kalyuzhnaya A.V., Boukhanovsky A. (2022). Automated evolutionary approach for the design of composite machine learning pipelines. Future Gener. Comput. Syst..

[46] nccr-itmo/FEDOT: Automated Modeling and Machine Learning Framework FEDOT. https://github.com/nccr-itmo/FEDOT.

[47] Probabilistic and Mean-Field Model of COVID-19 Epidemics with User Mobility and Contact Tracing | Semantic Scholar. https://www.semanticscholar.org/paper/Probabilistic-and-mean-field-model-of-COVID-19-with-Akian-Ganassali/9c8b962fb4ee58cb5cc3c25cbb29cbc30e2d583b.

[48] Alguliyev R., Aliguliyev R., Yusifov F. (2020). Graph modelling for tracking the COVID-19 pandemic spread. Infect. Dis. Model..

[49] Vepa A., Saleem A., Rakhshan K., Daneshkhah A., Sedighi T., Shohaimi S., Omar A., Salari N., Chatrabgoun O., Dharmaraj D. (2021). Using Machine Learning Algorithms to Develop a Clinical Decision-Making Tool for COVID-19 Inpatients. Int. J. Environ. Res. Public Health.

[50] Mihaljević B., Bielza C., Larrañaga P. (2021). Bayesian networks for interpretable machine learning and optimization. Neurocomputing.

